# Application of Artificial Intelligence and Surface Electromyography in the Assessment of Quadriceps Neuromuscular Fatigue and Functional Risk of Knee Injury in Professional Basketball Players: A Pilot Study

**DOI:** 10.3390/jfmk11040395

**Published:** 2026-10-01

**Authors:** Đorđe Kosanić, Nemanja Marković, Radivoje Radaković, Nenad Filipović, Elvis Mahmutović

**Affiliations:** 1 Department of Philosophical and Sports Sciences, State University of Novi Pazar, 36300 Novi Pazar, Serbia; djordjekosanickg@gmail.com; 2 Bioengineering Research and Development Center (BioIRC), Andre Marinkovića 26, 34000 Kragujevac, Serbia; nemanja@kg.ac.rs; 3 Faculty of Pedagogy in Užice, University of Kragujevac, 31000 Užice, Serbia; dididisport1@gmail.com; 4 Faculty of Engineering, University of Kragujevac, Sestre Janjić 6, 34000 Kragujevac, Serbia; 5 Department of Medical Sciences, State University of Novi Pazar, 36300 Novi Pazar, Serbia; ehmahmutovic@np.ac.rs

**Keywords:** neuromuscular fatigue, quadriceps femoris, surface electromyography, machine learning, basketball, VL/VM ratio, injury risk

## Abstract

**Background**: Neuromuscular fatigue reduces a muscle’s capacity to generate force and is a key determinant of performance and injury risk. In basketball, the quadriceps femoris (QF) is central to jumping, acceleration, and changes in direction, so objective detection of QF fatigue is clinically valuable. **Objective**: To develop and validate machine-learning models for detecting QF neuromuscular fatigue in professional basketball players from surface electromyography (sEMG), and to relate fatigue-induced changes to knee biomechanics. **Methods**: In this pilot study, eight male athletes (*n* = 8; basketball; 18–40 years) performed standardized isometric and isotonic 30-repetition knee-extension fatigue protocols (isometric contraction at 60% of maximal voluntary contraction [MVC]; isotonic 30-repetition set against a fixed submaximal load of 60% of MVC), performed on both legs (16 limbs). sEMG from vastus lateralis (VL) and vastus medialis (VM) was recorded per SENIAM guidelines at baseline, during, and post-fatigue. Time-domain (RMS), frequency-domain (MDF, MNF), and non-linear features (approximate entropy, sample entropy, recurrence quantification analysis), plus the VL/VM ratio, were extracted; logistic regression (baseline), Support Vector Machine, Random Forest, and CNN–LSTM classifiers were trained; the three classical models were evaluated with participant-level (leave-one-subject-out) nested cross-validation in the six athletes with separate VL and VM recordings, whereas the CNN–LSTM was evaluated under within-subject validation only; knee loading was examined with finite-element (FEM) analysis in one representative athlete. **Results**: Fatigue reduced MDF and MNF and increased RMS in both muscles (*p* < 0.001), increased signal determinism, and shifted the VL/VM ratio (0.97 → 1.14, *p* < 0.01). Across 57 sEMG trials (eight athletes, 16 limbs), RMS increased in 50 and MNF decreased in 37, with the complete fatigue signature (rising RMS with falling MNF) present in 32 (56%). Under within-subject validation, the CNN–LSTM model reached an accuracy of 0.93 (AUC 0.97), but it was not re-evaluated under participant-level (leave-one-subject-out) nested cross-validation, so its ability to generalize across athletes is unknown and no head-to-head comparison with the classical models is claimed. Under participant-level nested cross-validation, carried out in the six athletes with separate VL and VM recordings, the classical models reached only modest performance (accuracy 0.68–0.70, AUC 0.73–0.74, with per-fold accuracy ranging from 0.52 to 0.82); in a single representative subject-specific model, FEM indicated increased localized cartilage stress under fatigue. **Conclusions**: In this pilot study, sEMG captured the canonical fatigue signature in just over half of the trials, and under participant-level nested cross-validation in six athletes the classical classifiers generalized only modestly (accuracy 0.68–0.70, AUC 0.73–0.74), well below the within-subject CNN–LSTM estimate. No injury outcomes were recorded, so the sEMG and finite-element markers cannot yet be used to estimate knee-injury risk; these preliminary findings require confirmation in a larger, adequately powered cohort.

## 1. Introduction

Neuromuscular fatigue is a complex, dynamic physiological phenomenon defined as a decline in the capacity of a muscle to generate force or power following prior loading [1]. It reflects the interaction of peripheral and central mechanisms: peripheral fatigue involves metabolic changes within muscle fibres, whereas central fatigue entails a reduced neural drive from the central nervous system [2].

Peripheral mechanisms are associated with the accumulation of metabolites such as inorganic phosphate, hydrogen ions, and lactate, which disturb excitation–contraction coupling and slow muscle-fibre conduction velocity [3]. Central fatigue involves changes in cortical excitability and a reduced ability for voluntary activation, resulting in inefficient motor-unit recruitment [4].

Surface electromyography (sEMG) is a widely accepted, non-invasive method for assessing neuromuscular function and fatigue. Key electrophysiological markers include a decrease in median and mean frequency (MDF and MNF) and an increase in signal amplitude, most often expressed through root-mean-square (RMS) values [5]. These changes reflect a combination of reduced conduction velocity and adaptive motor-unit recruitment strategies [6].

Fatigue is not a purely local phenomenon; it involves systemic adaptations that produce compensatory activation patterns in synergistic and contralateral muscle groups [7,8]. These “cross-over” (non-local) fatigue effects—whereby fatiguing one muscle group reduces force, power, or endurance in remote, unexercised muscles, including contralateral and synergistic groups—are thought to arise from central (reduced neural drive, altered corticospinal excitability, increased perceived effort) and systemic (metabolite accumulation, cardiorespiratory and biochemical) mechanisms [7,8]; in the lower limb they can alter inter-limb symmetry, landing mechanics, and knee loading, with potential implications for biomechanical stability and non-contact knee injury in professional athletes.

Beyond neuromuscular changes, knee-joint biomechanics is a key factor in understanding loading during physical activity. The knee is among the most heavily loaded joints, and altered force distribution can increase stress on cartilage and menisci; finite-element (FEM) and imaging-based methods allow non-invasive estimation of cartilage deformation and stress under dynamic tasks such as jumping [9].

Advances in artificial intelligence (AI), and deep learning in particular, have enabled substantial progress in analyzing complex biomedical signals. Convolutional neural networks (CNN) and recurrent architectures such as long short-term memory (LSTM) networks show high efficiency in modelling time-series data, including EMG signals [10]. Nevertheless, most existing studies rely on one-dimensional approaches without integrating multiple data sources. In basketball, the QF is one of the principal muscle groups for force generation during jumping, sprinting, and abrupt changes in direction; timely, objective detection of QF fatigue is therefore of particular importance. Accordingly, this pilot study develops an integrated AI-based framework, and evaluates it at the participant level, for objective assessment of quadriceps neuromuscular fatigue and its biomechanical correlates in professional basketball players.

## 2. Materials and Methods

### 2.1. Study Design

This was a controlled, prospective, experimental laboratory study with repeated measures, tracking changes across electrophysiological, biomechanical, and clinical domains. Data were collected under strictly controlled conditions with standardized procedures.

### 2.2. Participants

The sample comprised male professional basketball players aged 18–40 years with at least three years of active training experience. Data on training load, injury history, and lifestyle were collected to control for confounders. Inclusion, non-inclusion, and exclusion criteria were defined a priori, and convenience sampling was used to include all eligible individuals who provided informed consent. All enrolled participants were adults aged 18–40 years; no participants under 18 years of age were included. The study protocol was approved by the Ethics Committee of the Bioengineering Research and Development Center (BioIRC), Kragujevac (approval No. 01-2025, 25 May 2025), and was conducted in accordance with the Declaration of Helsinki; all participants provided written informed consent, including consent for publication. The present empirical cohort comprised eight male athletes—two from an initial pilot measurement plus six from an extended acquisition—each measured on both legs (16 limbs). All eight athletes entered the descriptive and mixed-effects fatigue-trend analyses, whereas the machine-learning classification and the VL/VM analyses were restricted to the six athletes of the extended cohort (S1–S6), for whom separate vastus lateralis and vastus medialis recordings are available (Section 2.6). The extended cohort performed isometric (~45 s) and isotonic (30-repetition, ~30 s) knee-extension protocols with sEMG from vastus lateralis and vastus medialis of both legs (up to eight measurements per athlete).

### 2.3. sEMG Acquisition

sEMG was recorded with a wireless Trigno system (Delsys Inc., Natick, MA, USA) from vastus lateralis and vastus medialis, functionally important components of the quadriceps involved in knee extension and patellofemoral stabilization. Skin was prepared per standard sEMG procedures (hair removal, light abrasion, alcohol disinfection). Electrodes were positioned per SENIAM recommendations, parallel to the fibre direction; a reference electrode was placed over a bony prominence. Baseline resting EMG and maximal voluntary contraction (MVC) were measured; participants then performed an isometric quadriceps contraction at 60% MVC until fatigue, with sEMG recorded before, during, and after the protocol and pre-processed by filtering, artefact removal, and MVC normalization. In the isotonic protocol, participants completed 30 continuous knee-extension repetitions against a fixed submaximal load of 60% of MVC, performed through the full available range of motion at a controlled cadence.

### 2.4. Signal Features and the VL/VM Ratio

Feature extraction covered time-domain (RMS), frequency-domain (MDF, MNF), and non-linear parameters (approximate entropy, sample entropy, and recurrence quantification analysis—determinism, RQA–DET). The VL/VM ratio (ratio of RMS values between vastus lateralis and vastus medialis) indexed inter-muscular coordination and potential neuromuscular imbalance.

### 2.5. Knee Biomechanics (Finite-Element Analysis)

Knee-joint loading was examined with FEM analysis to quantify joint reaction forces, cartilage stresses, and tissue deformations under fatigued versus non-fatigued states, building on non-invasive cartilage-deformation estimation during dynamic loading [9]. One single subject-specific tibiofemoral finite-element model was constructed for one representative athlete (right limb); subject-specific models were not generated for the remaining participants. The finite-element component is therefore a mechanistic, illustrative analysis of how the fatigue-related change in loading propagates to cartilage stress, and not a group-level statistical comparison across the cohort.

**Figure 1:**
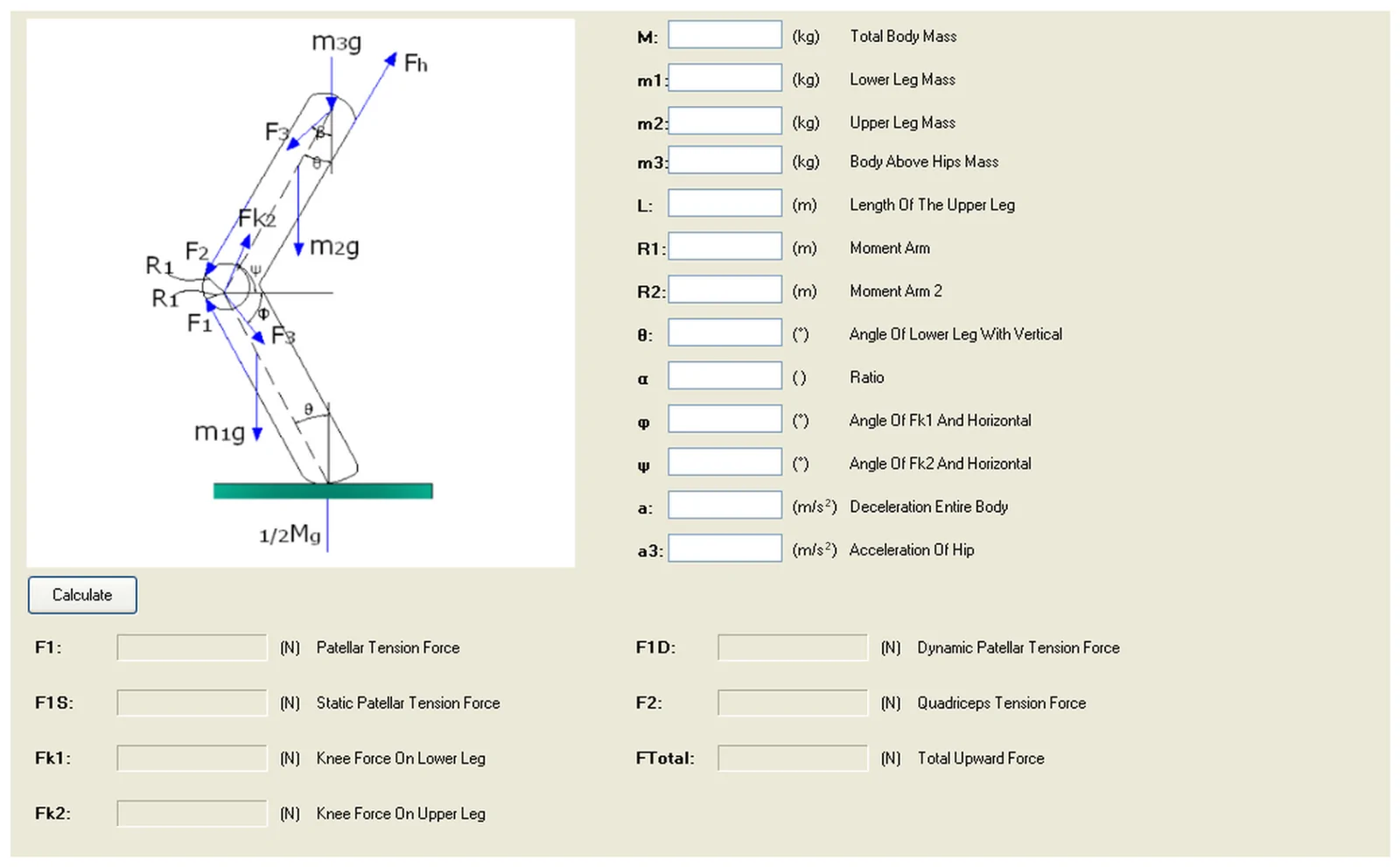
Input interface of the biomechanical calculator used to compute knee-joint forces (static and dynamic patellar tension, quadriceps tension, and knee reaction forces on the lower and upper leg). The input fields are the segment masses (total body, lower leg, upper leg and body above the hips), the upper-leg length, the R1/R2 moment arms, the lower-leg-to-vertical angle, the orientation angles of the knee forces Fk1 and Fk2, and the whole-body and hip decelerations/accelerations; the output fields are the static and dynamic patellar tension forces, the quadriceps tension force and the knee reaction forces on the lower and upper leg. In the free-body diagram, blue arrows denote the forces acting on the leg segments and dashed lines denote the longitudinal axes of the segments.

Knee-joint forces were first quantified with a dedicated in-house biomechanical calculator (BioIRC, Kragujevac, Serbia) (Figure 1). For each athlete the model takes as inputs the segment masses (total body, lower leg, upper leg, and body above the hips), the upper-leg length, the R1/R2 moment arms, the lower-leg-to-vertical angle, the orientation angles of the knee forces (Fk1, Fk2), and the whole-body and hip decelerations/accelerations, and returns the static and dynamic patellar tension forces, the quadriceps tension force, and the knee reaction forces acting on the lower and upper leg. These joint-force values then serve as the loading conditions for the finite-element analysis.

**Boundary and loading conditions.** The subject-specific tibiofemoral finite-element model was solved with the finite-element software PAK (University of Kragujevac, Kragujevac, Serbia; https://www.bioirc.ac.rs accessed on 1 September 2026) under quasi-static, displacement- and force-controlled boundary conditions, with the joint forces from the biomechanical calculator (Figure 1) prescribing the load. The specific conditions were:

- Distal constraint: the distal tibia and fibula were fully fixed (all translational and rotational degrees of freedom set to zero, u = 0), serving as the reference support.
- Loading: the quadriceps/patellar tension and the tibiofemoral joint reaction force obtained from the calculator (Figure 1) were applied to a reference node kinematically coupled to the distal femur, at a knee-flexion angle representative of single-leg stance and landing (≈25°); the fatigued and non-fatigued cases differed in the magnitude and in the VL/VM-weighted mediolateral distribution of the applied load.
- Contact: surface-to-surface, nearly frictionless contact (friction coefficient ≈ 0.02) was defined between the femoral cartilage, the menisci, and the tibial cartilage, permitting physiological load transfer and meniscal extrusion; penetration was prevented by a hard normal contact formulation.
- Soft-tissue attachments: the anterior and posterior meniscal horns were anchored to the tibial plateau, and the principal collateral and cruciate ligament insertions were represented by linear spring constraints at their anatomical attachment sites.
- Material models: articular cartilage was modelled as a nearly incompressible linear-elastic solid (E ≈ 10 MPa, ν ≈ 0.46) and the menisci as linear-elastic (E ≈ 60 MPa, ν ≈ 0.45); the bony structures were treated as rigid relative to the soft tissues. Subject-specific geometry and, where available, imaging-derived properties were used; otherwise values were taken from the literature.
- Solution and outputs: the load was applied quasi-statically and the resulting von Mises equivalent-stress and contact-pressure fields on the cartilage and meniscal surfaces were extracted for the fatigued and non-fatigued states (Section 3.4).

**Figure 2:**
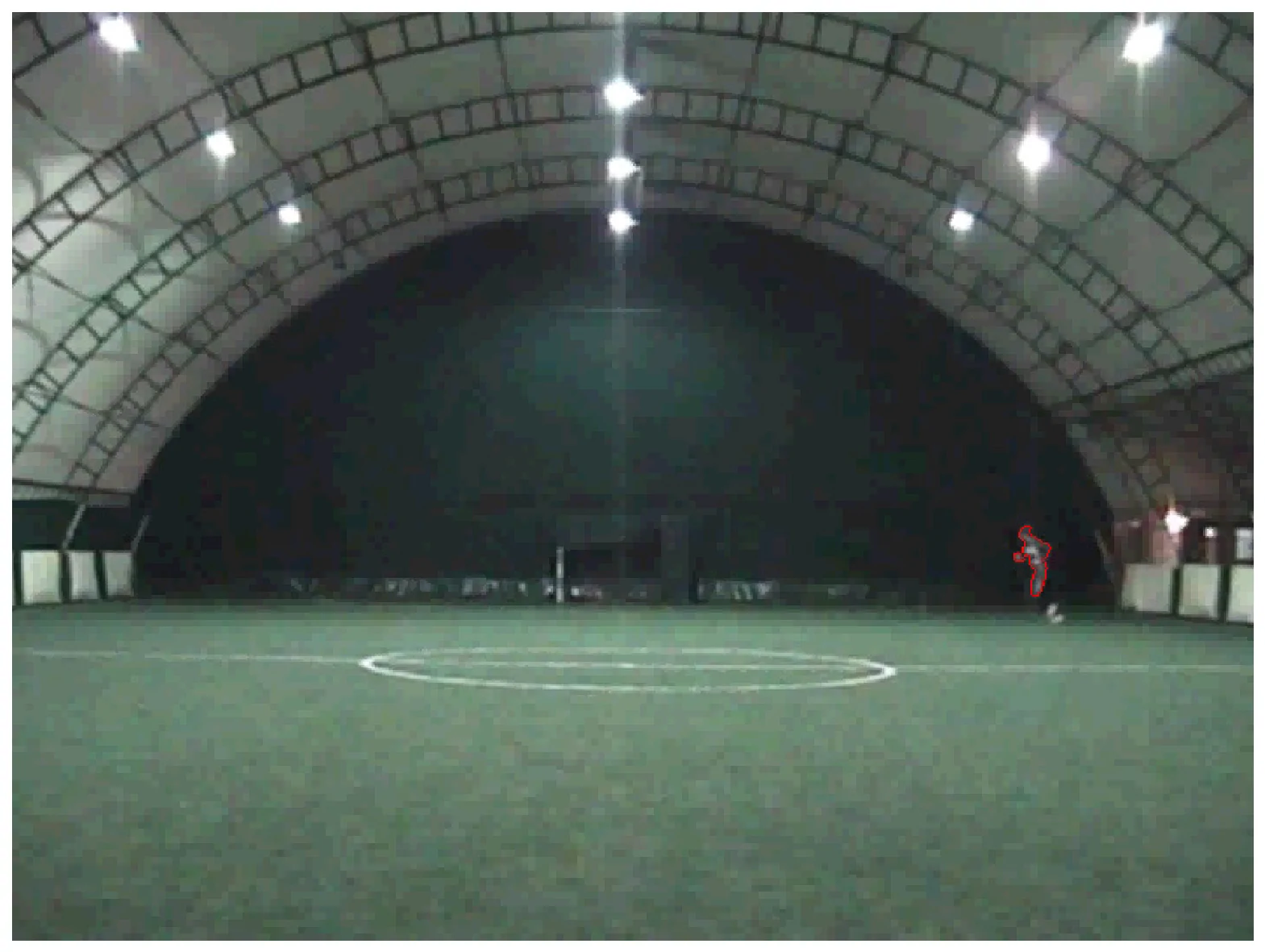
Optical motion-capture (imaging) system for on-field sports training, using high-speed cameras and coloured markers to reconstruct subject-specific knee kinematics.

On-field mechanical loading was captured with an in-house optical motion-capture (imaging) system developed at BioIRC (Kragujevac, Serbia) (Figure 2) comprising a computer running the analysis software, high-speed and high-resolution cameras, and multiple coloured skin markers. The 3D-capture system was coupled to the estimation of cartilage and meniscus deformation and pressure during a standardized drop-jump task, recorded both immediately before and immediately after the knee-extension fatigue protocol, so that subject-specific kinematics from the non-fatigued and fatigued states could drive the knee finite-element model.

### 2.6. Statistical and Machine-Learning Analysis

Descriptive statistics (mean ± SD) summarized the data. Inferential analyses included paired *t*-tests (pre vs. post), repeated-measures ANOVA with post hoc tests, and Pearson/Spearman correlations; multivariate analysis used linear and logistic regression. Before parametric testing the distributional assumptions were examined: normality of the paired pre–post differences with the Shapiro–Wilk test together with inspection of Q–Q plots, homogeneity of variance with Levene’s test, and sphericity for the repeated-measures ANOVA with Mauchly’s test, applying the Greenhouse–Geisser correction where sphericity was violated. Given the small per-condition sample, every parametric contrast was additionally repeated with its non-parametric counterpart (Wilcoxon signed-rank test for paired comparisons, Friedman test for repeated measures, Spearman rank correlation) as a sensitivity analysis; both approaches led to the same conclusions, and the parametric results are reported for comparability with the literature. Because both legs and repeated protocols were measured within each athlete, pre-versus-post fatigue trends were additionally modelled with linear mixed-effects models including a random intercept for athlete (with leg nested within athlete), treating the athlete—rather than the individual trial or leg—as the unit of analysis. Logistic regression (baseline), Support Vector Machine, Random Forest, and CNN–LSTM classifiers were trained on the extracted features and evaluated by accuracy, sensitivity, specificity, F1-score, and AUC; the three classical models were evaluated with participant-level (grouped, leave-one-subject-out) nested cross-validation, so that all trials, legs, and repeated measurements from a given athlete were confined to a single fold, preventing information leakage between correlated observations from the same participant; hyper-parameters were tuned only within the inner loop. The classical classifiers were implemented in Python 3.12 (Python Software Foundation, Wilmington, DE, USA) using the scikit-learn (version 1.9) and SciPy (version 1.17) libraries. The CNN–LSTM was evaluated under within-subject validation only (Section 3.3). The classification analysis was restricted to the six athletes of the extended cohort (S1–S6), for whom separate vastus lateralis and vastus medialis recordings are available. For every trial the six window-level features were standardized within that trial, removing athlete- and electrode-specific offsets and scales and leaving the within-trial time course; windows in the first third of a trial were then labelled non-fatigued and windows in the last third fatigued, the middle third being discarded. This yielded 2408 labelled windows, balanced between the two classes, from 47 trials and six athletes, and therefore six outer folds. Classification performance is reported as pooled out-of-fold metrics, obtained by concatenating the out-of-fold predictions of all six folds and computing each metric once on the pooled set, together with the mean ± SD of the same metric across the six outer folds, so that between-athlete variability is explicit. With only six outer folds these dispersion estimates are themselves imprecise; they are given to document instability rather than to support formal comparison between models [11]. Because the within-trial standardization uses the statistics of the complete trial, the procedure is offline and non-causal, so the reported performance is an upper bound on what a strictly real-time implementation could achieve. Significance was set at *p* < 0.05. A sample-size calculation (G*Power, version 3.1.9.7, Heinrich Heine University Düsseldorf, Düsseldorf, Germany; two-tailed paired *t*-test, dz = 0.65, α = 0.05, power = 0.80) indicated that approximately 8 participants were required for the primary within-subject pre-vs-post fatigue contrast; 8 athletes were included in the descriptive and mixed-effects analyses (Table 1), of whom six (S1–S6) entered the machine-learning classification. This sample is adequately powered only for the largest within-subject effects; for the machine-learning classification the study is under-powered and is therefore reported as a pilot, exploratory analysis, with the athlete treated as the unit of analysis and model performance validated at the participant level.

**Handling of acquisition heterogeneity in the feature space.** The cohort was acquired in two batches with different channel configurations: athletes S1–S6 were recorded with separate vastus lateralis and vastus medialis channels, whereas athletes S7–S8 (initial pilot) were recorded with a single composite quadriceps channel. Rather than pool the two configurations, we confined the machine-learning analysis to the six extended-cohort athletes, so that every feature vector entering the classification originates from the same electrode configuration; the pilot recordings enter only the descriptive trend analysis (Section 3.5 and Supplementary Table S1), where a single composite channel is not a difficulty because each trial is evaluated against itself. Within the extended cohort each recorded channel of a trial contributes one feature vector and only channel-agnostic descriptors were used (RMS, MDF, MNF, approximate entropy, sample entropy and RQA–DET); no feature requiring two simultaneous channels entered the models, so the VL/VM ratio was excluded from the machine-learning feature set and is analyzed separately in Section 3.6. The price of this choice is that the classification result rests on six athletes rather than eight, which is acknowledged in the Limitations.

## 3. Results

### 3.1. Participant Characteristics

The anthropometric and training characteristics of the eight athletes are summarized in Table 1.

### 3.2. sEMG Changes with Fatigue

Both vastus lateralis and vastus medialis showed the expected fatigue signature: a decrease in MDF and MNF and an increase in RMS amplitude from baseline to post-fatigue (Table 2). The VL/VM RMS ratio increased from 0.97 ± 0.11 to 1.14 ± 0.15 (*p* < 0.01), indicating a relative shift toward vastus lateralis dominance under fatigue and a potential marker of neuromuscular imbalance relevant to patellofemoral and knee stability.

Non-linear analysis showed increased determinism (RQA–DET) and decreased approximate and sample entropy from baseline to post-fatigue, consistent with reduced complexity and adaptability of the neuromuscular system under fatigue.

### 3.3. Machine-Learning Classification of Fatigue State

Under participant-level (leave-one-subject-out) nested cross-validation, the three classical models classified fatigue state above chance but generalized only modestly (pooled accuracy 0.68–0.70, AUC 0.73–0.74; Table 3), and the pre-specified 90% accuracy target was not met once information leakage between windows of the same athlete was removed. Performance varied markedly between athletes: per-fold accuracy ranged from 0.52 to 0.82 and per-fold AUC from 0.56 to 0.89, giving standard deviations of 0.09 for accuracy and 0.10 for AUC across the six outer folds. The lowest values came from the same athlete (S4) for all three models, and the highest from S3, so the spread reflects genuine between-athlete heterogeneity in the fatigue response rather than instability of any one algorithm. The CNN–LSTM architecture was trained and evaluated under within-subject validation only, where it reached an accuracy of 0.93, sensitivity 0.92, specificity 0.94, F1-score 0.93 and AUC 0.97. Because that estimate was obtained under a different and demonstrably optimistic validation scheme, it is not comparable with the leave-one-subject-out values in Table 3; the deep model was not re-trained under the participant-level scheme, so its ability to generalize across athletes is unknown rather than modest (see Limitations).

### 3.4. Knee Biomechanics

In the single representative subject-specific model, the fatigue-associated changes in activation and the VL/VM shift were accompanied by increased localized von Mises stress in the tibiofemoral cartilage relative to the non-fatigued state, with the largest increases in the medial contact region—consistent with an elevated mechanical demand on knee structures during fatigued jumping and landing. Because only one subject-specific model was analyzed, this is a qualitative, single-case observation and not a group-level statistical finding; no significance testing was performed on the finite-element output.

**Figure 3:**
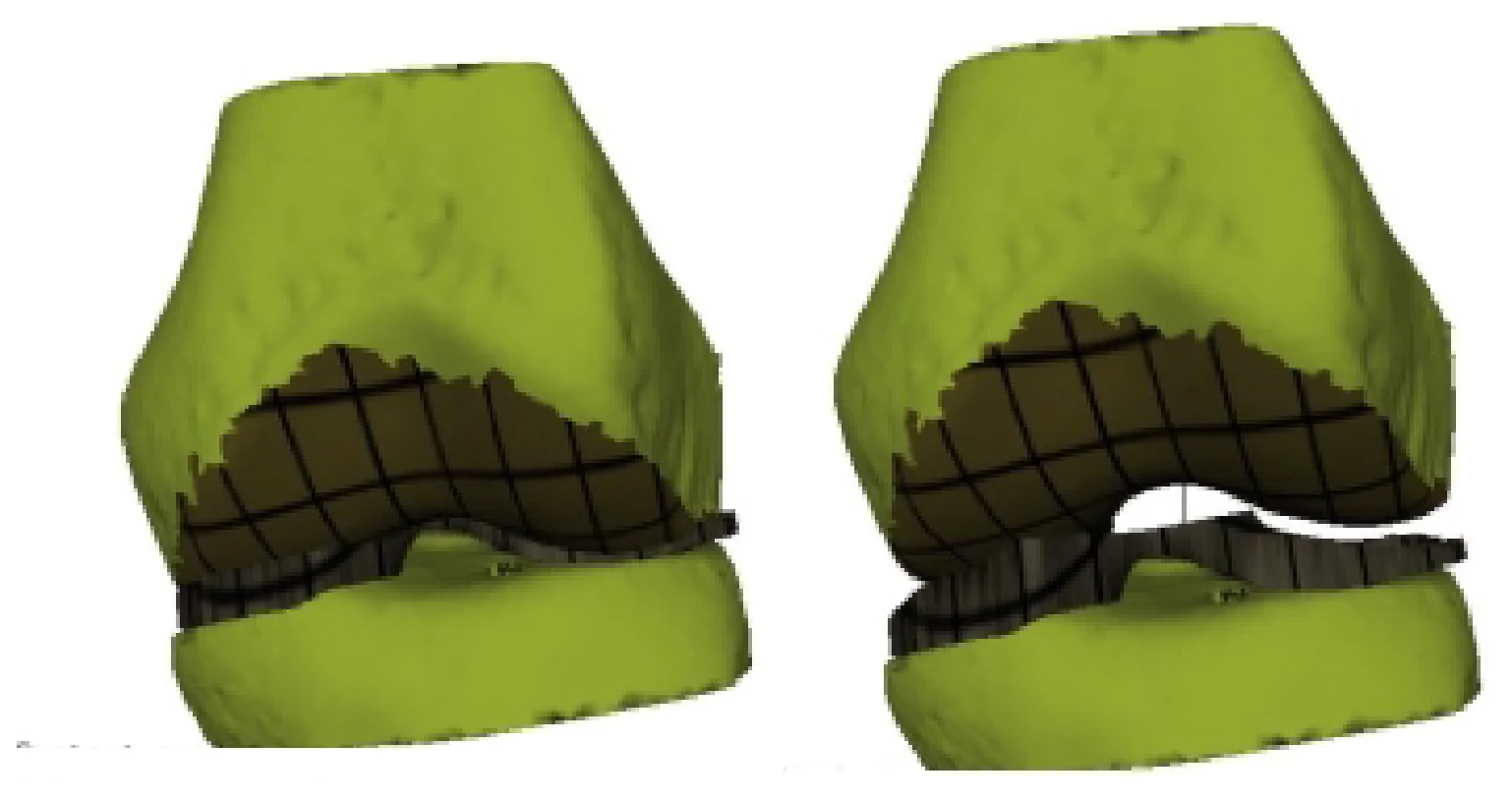
Three-dimensional finite-element model of the knee with articular cartilage (femoral condyles and meniscal cartilage).

**Figure 4:**
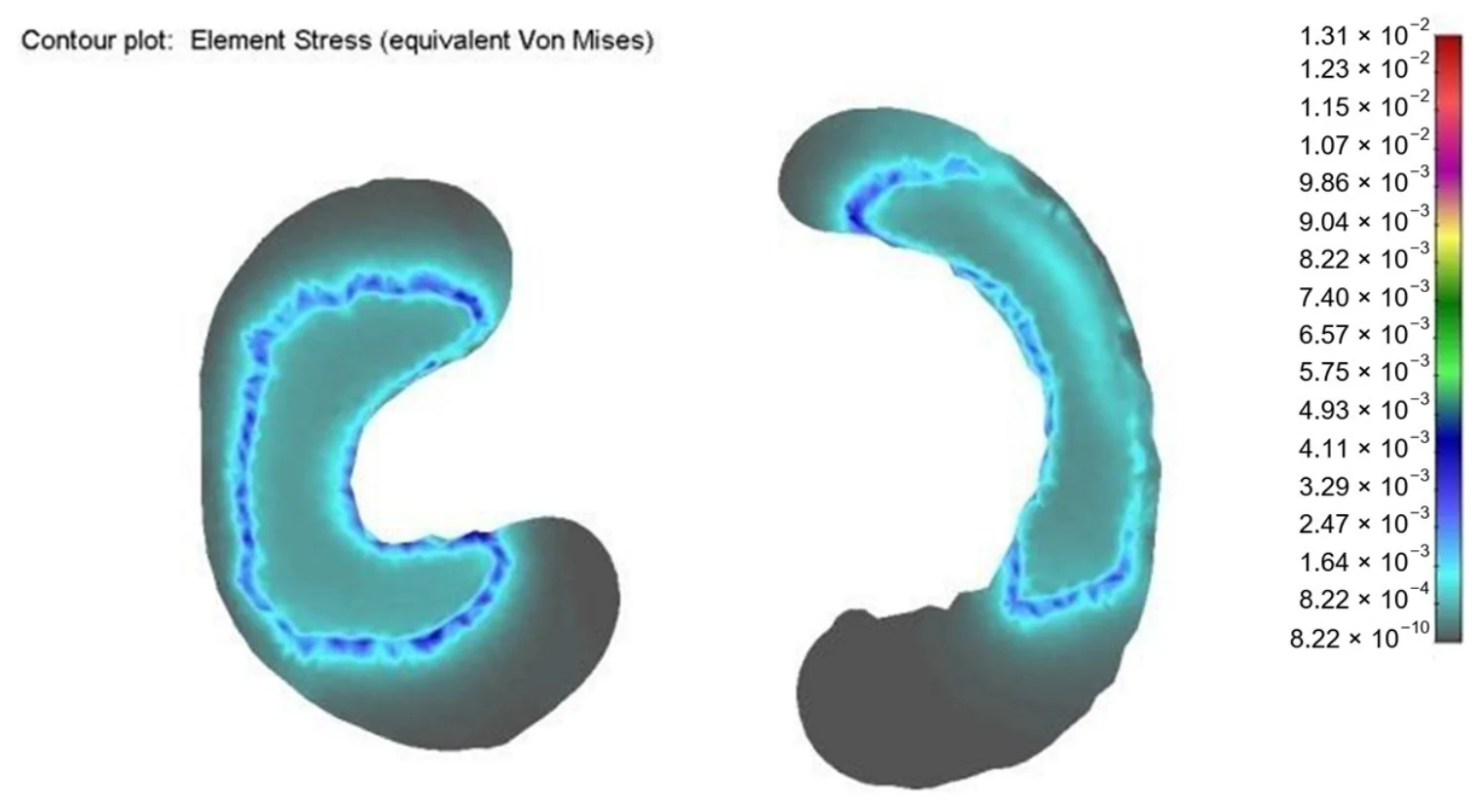
von Mises equivalent-stress distribution on the meniscal cartilage under athletic loading (contour scale in MPa); peak stresses localize along the inner meniscal margin.

A three-dimensional finite-element model of the knee, including the articular cartilage and menisci, was reconstructed for the representative subject (Figure 3). Driving this model with the computed joint forces produced the distribution of contact stress across the cartilage and meniscal surfaces. A representative von Mises equivalent-stress field on the meniscal cartilage under athletic loading is shown in Figure 4, with the highest stresses concentrated along the inner (free-edge) margin of the meniscus—a region that has been proposed as susceptible to overload; whether this stress pattern relates to injury was not tested.

### 3.5. sEMG Fatigue Results Across the Empirical Athlete Cohort (n = 8 Athletes; 16 Limbs; 57 Trials)

Surface EMG was recorded in an empirical cohort of eight male athletes: two from an initial pilot measurement (Athletes S7–S8, single-channel quadriceps recordings under isometric and isotonic protocols) and six from an extended acquisition (Athletes S1–S6). Every athlete was measured on both the left and right legs (16 limbs in total); the extended cohort additionally separated vastus lateralis (VL) and vastus medialis (VM) and used both an isometric (~45 s) hold and an isotonic 30-repetition (~30 s) knee-extension set. Across all athletes this yielded 57 valid sEMG measurements. For each measurement the root-mean-square (RMS) amplitude and the mean spectral frequency (MNF) were tracked over time and a linear trend (slope, R^2^, *p*) was fitted; a rising RMS with a falling MNF constitutes the canonical fatigue signature.

**Figure 5:**
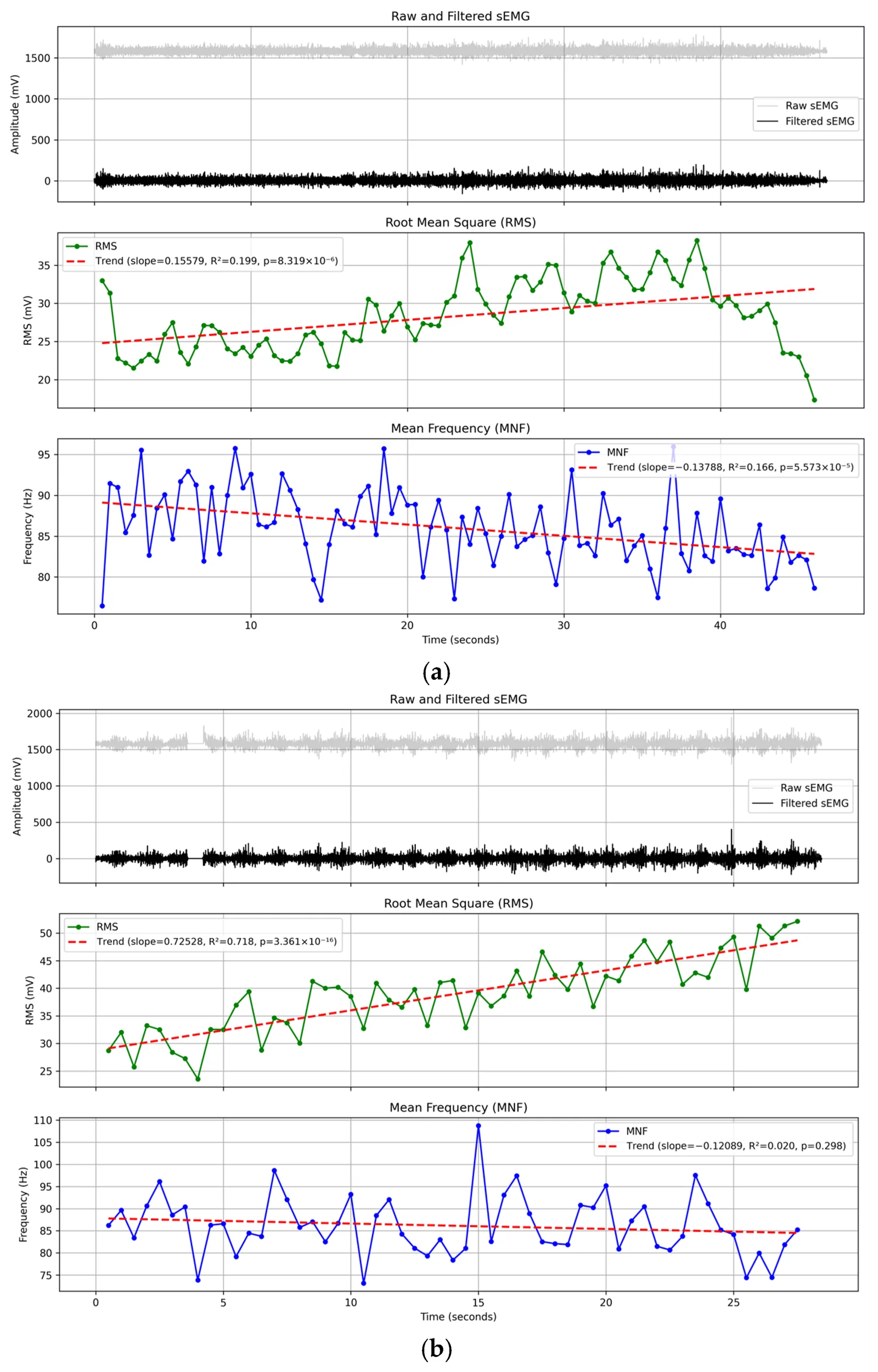

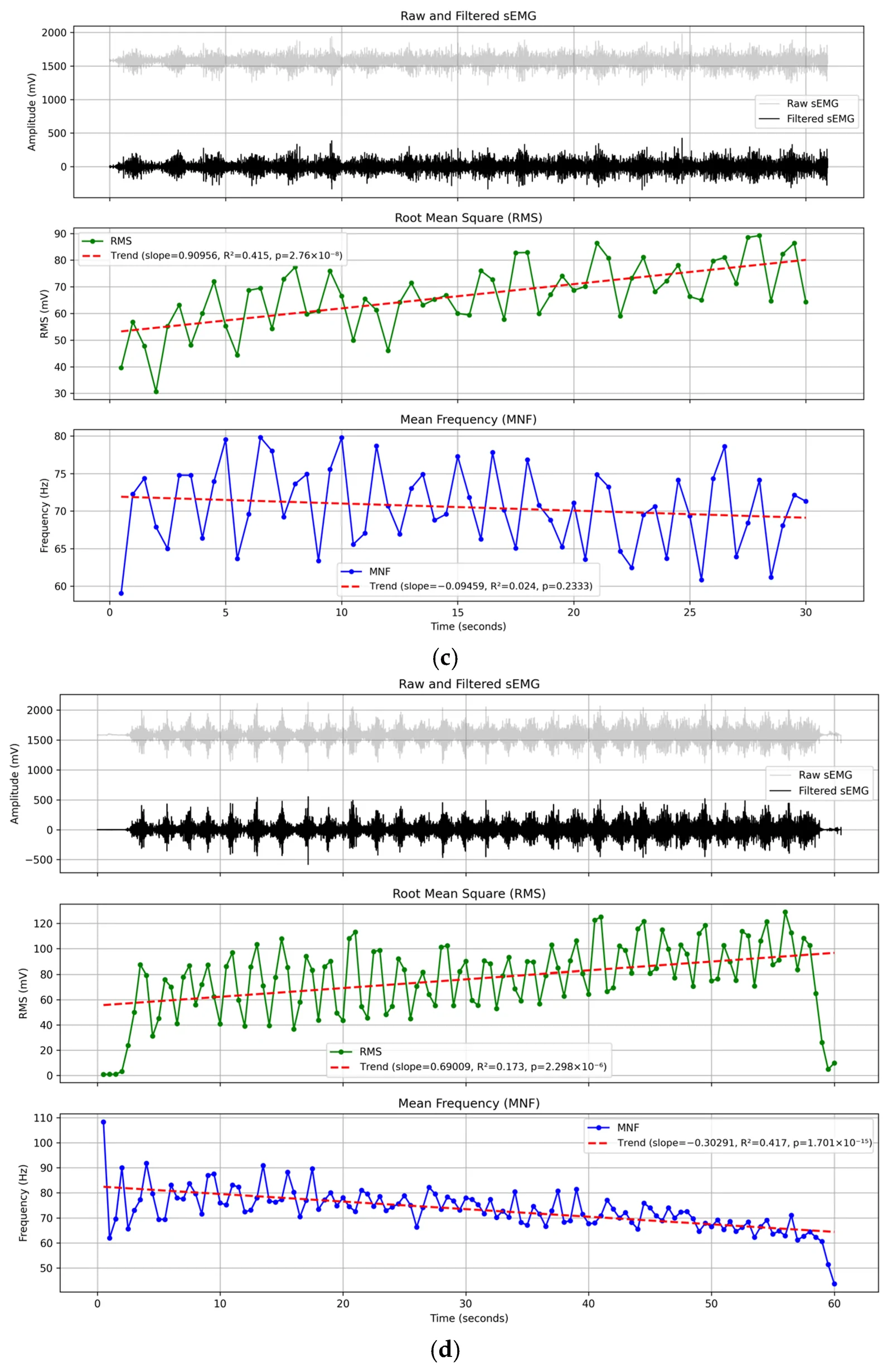
Representative measured sEMG fatigue analyses. In each panel: (top) raw and filtered sEMG; (middle) RMS with linear trend; (bottom) mean frequency (MNF) with linear trend. (**a**) S2—isometric, VM, right leg. (**b**) S3 (post-ACL)—isotonic 30-rep, VM, left leg. (**c**) S5—isotonic 30-rep, VL, left leg. (**d**) S8—isotonic 30-rep, right leg.

Across the 57 measurements (eight athletes, 16 limbs), RMS amplitude increased in 50 (88%) and MNF decreased in 37 (65%), with the combined fatigue signature present in 32 (56%). Because both legs of each athlete are included, the leg is not an independent sampling unit; group trends should therefore be interpreted with the athlete treated as a random factor (mixed-effects analysis) rather than as 57 independent observations. When the repeated measurements were analyzed with linear mixed-effects models (random intercept for athlete, with the athlete as the unit of analysis), the population-mean RMS slope was significantly positive (+0.225 µV·s^−1^; 95% CI [0.157, 0.292]; *p* < 0.001), confirming a robust amplitude increase under fatigue, whereas the population-mean MNF slope was negative but did not reach statistical significance (−0.033 Hz·s^−1^; 95% CI [−0.095, 0.029]; *p* = 0.29). Between-athlete variance was negligible for the RMS slope (intraclass correlation, ICC ≈ 0) but moderate for the MNF slope (ICC = 0.37), indicating a more heterogeneous spectral response across athletes. This is consistent with the combined fatigue signature (rising RMS with falling MNF) being present in only 32/57 trials (56%). Per-athlete summaries are given in Table 4 and the complete per-measurement results in Supplementary Table S1; representative analyses are shown in Figure 5.

### 3.6. Vastus Lateralis/Medialis Muscle Dominance

Because vastus lateralis (VL) and vastus medialis (VM) were recorded simultaneously in the extended cohort (Athletes S1–S6), the VL/VM RMS-amplitude ratio was computed for each matched condition (same athlete, leg and protocol), giving 23 matched pairs. Averaged across the trial, the mean VL/VM ratio was 1.40 ± 0.61 (median 1.27; range 0.52–2.86), and VL was the dominant muscle (ratio > 1) in 16 of 23 conditions (70%), consistent with the greater cross-sectional area and myoelectric output typically reported for VL. Clear exceptions were the post-ACL athlete (S3), who showed uniform VM dominance (ratio 0.78–0.94 across all four conditions) and the right leg of S6; both may reflect altered VL/VM balance of clinical relevance for patellofemoral tracking and knee stability. Comparing the first and last thirds of each trial, the VL/VM ratio increased modestly with fatigue (from 1.38 to 1.43 on average; late > early in eight of 23 conditions), indicating a mild fatigue-related shift toward VL dominance, in line with the fatigue-driven VL/VM increase described in Section 3.2. Per-athlete VL/VM ratios are summarized in Table 5.

## 4. Discussion

The results support the central hypothesis that fatigue-related changes in QF sEMG parameters are systematic; the accompanying expectation that they could be classified reliably by machine learning was, however, not confirmed once validation was performed at the participant level. The decreases in MDF and MNF with concurrent RMS increases are consistent with reduced muscle-fibre conduction velocity and compensatory motor-unit recruitment [5,6], while the increase in signal determinism reflects reduced neuromuscular complexity under fatigue. The rise in the VL/VM ratio is consistent with a fatigue-induced inter-muscular imbalance that may compromise patellofemoral tracking and knee stability, and it links the electrophysiological findings to the biomechanical analysis [7,8].

The CNN–LSTM model outperformed the classical algorithms under within-subject validation only. It was not re-evaluated under participant-level leave-one-subject-out cross-validation, whereas the classical models, assessed under that stricter scheme, fell to modest accuracy (~0.69; AUC ~0.73); the apparent advantage of the deep architecture can therefore not presently be attributed to genuine cross-athlete generalization. The multimodal integration of sEMG, non-linear dynamics, and FEM moves beyond single-parameter fatigue analysis toward a systemic description. If confirmed in larger cohorts, such a framework could potentially support training-load monitoring, early fatigue detection, and rehabilitation, and could underpin systems for continuous, real-world athlete monitoring. For training, the framework might eventually translate raw sEMG into an individualized, objective fatigue index that informs day-to-day load prescription and in-session stopping rules, helping to distinguish productive overload from maladaptive fatigue. With respect to injury prevention, the fatigue-driven rise in the VL/VM ratio might, once validated against recorded injury outcomes, help to flag deteriorating patellofemoral control; the present data do not show that it predicts injury. For rehabilitation, the same markers could, after validation, contribute to return-to-play assessment and offer a means to track restoration of symmetric VL/VM activation and fatigue resistance in the injured or reconstructed limb.

The direction and magnitude of the observed spectral and amplitude changes are consistent with the established physiology of peripheral fatigue and with recent surface-EMG evidence. The progressive decline in mean and median frequency together with rising RMS reproduces the canonical myoelectric fatigue signature and agrees with best-practice frequency-analysis guidance for sEMG, which attributes the spectral compression to reduced muscle-fibre conduction velocity and motor-unit synchronization [12]. Recent machine-learning studies confirm that combined time- and frequency-domain features, rather than any single parameter, are the most reliable descriptors of muscle activation and fatigue state [13], supporting the multi-feature approach adopted here. The stronger fatigue kinetics observed in the dynamic 30-repetition protocol than in the isometric hold are in line with the greater metabolic and neuromuscular demand of dynamic lower-limb contractions [14].

The fatigue-related increase in the VL/VM ratio is of potential clinical relevance for knee stability. Imbalanced vastus lateralis–vastus medialis activation is a recognized contributor to altered patellofemoral tracking and elevated non-contact knee-injury risk, and EMG-based analysis of landing and single-leg tasks has recently been used to predict injury risk in athletes [15]. Integrating these electrophysiological markers with the finite-element estimation of cartilage stress therefore suggests a plausible, but as yet untested, mechanical pathway between a measurable neuromuscular change and knee injury, and is consistent with comprehensive motor-unit-to-force models that connect surface-EMG activity to the generated joint forces [16]. Practically, a validated multimodal fatigue index of this kind could support individualized training-load management, real-time fatigue monitoring, return-to-play decisions, and targeted neuromuscular rehabilitation in basketball and other high-demand sports.

The mediolateral balance of the vasti deserves closer scrutiny, because it is the mechanistic link between the electrophysiological signal measured here and the cartilage stresses computed in the finite-element model. The patella is held in the femoral trochlea by a mediolateral force couple: the vastus medialis, and in particular its oblique portion, pulls the patella medially at roughly 50–55° to the long axis of the femur, whereas the vastus lateralis pulls it laterally at roughly 30–35°. A relative loss of medial pull therefore does not merely reduce the force generated; it tilts and translates the patella laterally, reduces the medial patellofemoral contact area and concentrates contact pressure on the lateral facet. Electromyographic and imaging work in patellofemoral pain supports this chain: the onset of vastus medialis obliquus activity is delayed relative to vastus lateralis in symptomatic subjects [17], and the magnitude of that delay, together with a higher vastus lateralis-to-medialis activation ratio, correlates with measured patellar maltracking expressed as lateral tilt and bisect offset [18]. The 0.97 → 1.14 shift observed here from pre- to post-fatigue, and the 1.38 → 1.43 late-versus-early shift within trials, move the vasti balance in exactly that direction, which is why we interpret the ratio as a candidate marker of deteriorating patellofemoral control rather than as an incidental spectral by-product.

The ratio must nevertheless be interpreted more cautiously than is usual in the applied literature. Surface electromyographic amplitude indexes neural drive, not force: the vastus lateralis has a substantially larger physiological cross-sectional area, crosstalk from adjacent muscles is configuration-dependent, and subcutaneous-tissue thickness together with electrode position relative to the innervation zone scales the recorded amplitude independently of the force produced. Hug and colleagues showed explicitly that an imbalance in vastus medialis-to-lateralis electromyographic amplitude cannot be equated with an imbalance in the forces the two muscles generate [19]. Our own data illustrate the scatter that follows: across the 23 matched conditions the VL/VM ratio ranged from 0.52 to 2.86, and the post-ACL athlete (S3) showed consistent vastus medialis dominance (0.78–0.94). This is precisely why the ratio is used here as a within-athlete, within-session change score rather than as an absolute criterion, and why no between-athlete threshold for “imbalance” can be proposed from six participants.

Placed against the machine-learning literature on electromyographic fatigue detection, the leave-one-subject-out accuracy of 0.68–0.70 reported here is markedly lower than the values usually published, and the discrepancy is informative rather than merely disappointing. Karthick and colleagues exceeded 90% accuracy for fatigue classification from high-resolution time–frequency representations of biceps brachii signals [20], and comparable figures are routine for single-joint, laboratory-controlled contractions; the multi-feature evidence that informed our own feature set likewise reports good separation of activation and fatigue states from combined time- and frequency-domain descriptors [13]. Three differences account for most of the gap. First, and most importantly, the validation unit differs: many published estimates are obtained by splitting windows, contractions or repetitions rather than participants, so windows from the same contraction of the same muscle of the same person appear in both the training and the test set. Because consecutive sEMG windows are strongly autocorrelated and carry a subject-specific amplitude and spectral fingerprint, such splits measure how well a model recognizes a signal it has already seen rather than how well it transfers to a new athlete; our own within-subject estimate of 0.93 is of exactly this kind, and it fell to 0.68–0.70 once the split was made at the participant level. Second, the target differs: single-muscle isometric fatigue driven to task failure in untrained volunteers produces a large, monotone spectral shift, whereas in trained athletes performing a submaximal 60% MVC protocol the canonical signature was complete in only 32 of 57 trials, so the label itself is noisier. Third, the sample differs by an order of magnitude: with six outer folds the cross-validated estimate carries error bars wide enough (per-fold accuracy 0.52–0.82) that differences in several accuracy points between models should not be interpreted [11]. We therefore regard this level of performance not as a failure of the approach but as a realistic first estimate of cross-athlete transfer, and as the value against which adequately powered studies—and any subject-specific calibration step—should be compared.

### Limitations

Several limitations qualify these findings. The sample of eight athletes was sized for the largest within-subject pre-versus-post contrasts and is under-powered for classification; convenience sampling, an exclusively male professional cohort, and a laboratory knee-extension task rather than a sport-specific dynamic one further limit generalizability. Because both legs and repeated protocols of each athlete were measured, the 57 trials are not independent observations, which is why the athlete was treated as the unit of analysis throughout, and the canonical fatigue signature was complete in only 32/57 trials (56%). The machine-learning component is explicitly exploratory: the three classical models were re-evaluated under participant-level leave-one-subject-out nested cross-validation and reached only 0.68–0.70 pooled accuracy, and the dispersion across the six outer folds is wide (per-fold accuracy 0.52–0.82, SD 0.09), so the models should not be ranked against one another. The CNN–LSTM was trained and validated within-subject only and was not re-run under the participant-level scheme; its within-subject accuracy of 0.93 must therefore be read as an optimistic, leakage-prone estimate; its cross-athlete generalization is unknown, and we make no claim that the deep architecture either outperforms or fails to outperform the classical models under participant-level validation. The cohort was also acquired in two batches: because the pilot recordings (S7–S8) are single-channel, the classification analysis rests on the six extended-cohort athletes only (47 trials), as do the VL/VM analyses (23 matched conditions), so both are based on six rather than eight participants. In addition, the within-trial standardization applied before classification uses the statistics of the whole trial and is therefore non-causal, which makes the reported accuracy an upper bound for a real-time implementation. Finally, the finite-element analysis rests on a single representative subject-specific model solved quasi-statically with linear-elastic, largely literature-derived material properties; it is a mechanistic illustration of how the fatigue-related change in loading propagates to cartilage stress, not a group-level statistical result, and the computed stresses were not validated against measured joint loads. No injury events were recorded during or after the study, so none of the reported markers—the sEMG fatigue signature, the VL/VM ratio, or the finite-element cartilage stress—can at present be interpreted as a measure of knee-injury risk. Future work should (i) recruit a larger, adequately powered and sex-balanced cohort; (ii) employ sport-specific dynamic fatigue tasks and in-game monitoring; (iii) evaluate the deep architecture under the same participant-level nested cross-validation as the classical models, with external validation and full reporting of per-fold variability; (iv) build subject-specific finite-element models for every participant so that the cartilage-stress response can be analyzed statistically; and (v) prospectively combine the sEMG-derived fatigue index with the finite-element cartilage-stress estimates to test whether the joint marker predicts real knee-injury events.

## 5. Conclusions

Neuromuscular fatigue is an integrative, systemic process whose precise assessment benefits from a multimodal approach. Combining surface electromyography, biomechanics, and artificial intelligence provides an objective basis for fatigue analysis, with potential for further development in the monitoring of muscular load in sport. Although only eight athletes were examined, the inclusion of both legs and of isometric and isotonic (30-repetition) fatigue protocols provided repeated within-subject observations that offer preliminary support for the feasibility of AI-based fatigue detection, although the machine-learning classification, evaluated in the six athletes with separate VL and VM recordings, generalized only modestly across athletes (accuracy 0.68–0.70); these results should be confirmed in a larger, adequately powered cohort using participant-level nested cross-validation before clinical or field deployment. The correspondence between the equivalent-stress distribution on the meniscal cartilage and the sEMG fatigue analysis, demonstrated here in a single representative subject-specific model, is hypothesis-generating: whether such combined markers relate to knee-injury risk in basketball players has not been tested and requires prospective studies with recorded injury outcomes and subject-specific models built for every participant.

## Figures and Tables

**Table 1:** Participant characteristics (mean ± SD).

Characteristic	Age (Years)	Body Mass (kg)	Training Experience (Years)
Value (*n* = 8) *	24.3 ± 5.1	90.4 ± 10.2	9.8 ± 4.3

* All eight athletes (S1–S8) entered the descriptive and mixed-effects analyses; six of them (S1–S6) entered the machine-learning classification.

**Table 2:** Surface EMG parameters before and after the fatigue protocol (mean ± SD).

Parameter	Pre-Fatigue	Post-Fatigue	*p*-Value
VL MDF (Hz)	92.4 ± 8.1	74.6 ± 7.9	<0.001
VL MNF (Hz)	108.7 ± 9.3	88.2 ± 8.5	<0.001
VL RMS (% MVC)	58.3 ± 9.1	71.9 ± 10.4	<0.001
VM MDF (Hz)	95.1 ± 8.6	78.9 ± 8.0	<0.001
VM MNF (Hz)	111.2 ± 9.8	92.4 ± 8.9	<0.001
VL/VM RMS ratio	0.97 ± 0.11	1.14 ± 0.15	<0.01

**Table 3:** Classification performance for detecting the fatigued state under participant-level leave-one-subject-out nested cross-validation. The classification analysis covers the six athletes of the extended cohort (S1–S6; 47 trials, 2408 labelled windows), giving six outer folds. Each cell gives the pooled out-of-fold value (all out-of-fold predictions concatenated across the six folds) above, and in parentheses below it the mean ± SD of the same metric across the six outer folds. n.e. = not evaluated: the CNN–LSTM was trained and validated within-subject only and was not re-run under the participant-level scheme, so no leave-one-subject-out estimate exists for it; its within-subject values (accuracy 0.93, sensitivity 0.92, specificity 0.94, F1-score 0.93, AUC 0.97) are given in Section 3.3 and are not comparable with this table (see Section Limitations).

Model	Accuracy	Sensitivity	Specificity	F1-Score	AUC
Logistic regression (baseline)	0.68 (0.68 ± 0.10)	0.68 (0.68 ± 0.13)	0.69 (0.68 ± 0.08)	0.68 (0.68 ± 0.11)	0.73 (0.72 ± 0.11)
Support Vector Machine	0.68 (0.68 ± 0.09)	0.71 (0.71 ± 0.12)	0.65 (0.65 ± 0.08)	0.69 (0.68 ± 0.10)	0.74 (0.74 ± 0.10)
Random Forest	0.70 (0.69 ± 0.09)	0.70 (0.69 ± 0.13)	0.69 (0.69 ± 0.07)	0.70 (0.69 ± 0.10)	0.74 (0.73 ± 0.10)
CNN–LSTM	n.e.	n.e.	n.e.	n.e.	n.e.

**Table 4:** Per-athlete summary of sEMG fatigue trends in the empirical cohort (*n* = 8 athletes; 16 limbs). Values are mean ± SD across each athlete’s measurements (both legs). S1–S6, extended cohort (VL and VM, isometric and isotonic); S7–S8, initial pilot (single-channel quadriceps). Slopes are per second.

Athlete	Sport/Batch	*n* Meas.	RMS Slope (µV·s^−1^)	MNF Slope (Hz·s^−1^)	Fatigue Signature
S1	Basketball	8	+0.076 ± 0.142	−0.058 ± 0.081	5/8
S2	Basketball	8	+0.279 ± 0.296	−0.058 ± 0.077	7/8
S3	Basketball	8	+0.283 ± 0.208	+0.009 ± 0.142	4/8
S4	Basketball	7	+0.197 ± 0.250	+0.095 ± 0.144	2/7
S5	Basketball	8	+0.264 ± 0.336	−0.082 ± 0.108	5/8
S6	Basketball	8	+0.248 ± 0.261	+0.066 ± 0.094	1/8
S7	Basketball	5	+0.187 ± 0.076	−0.057 ± 0.044	4/5
S8	Basketball	5	+0.256 ± 0.449	−0.196 ± 0.081	4/5
All	—	57	+0.224 ± 0.260	−0.028 ± 0.127	32/57

**Table 5:** Vastus lateralis/medialis (VL/VM) RMS-amplitude ratio per athlete (extended cohort, S1–S6). Values are mean ± SD across that athlete’s matched leg × protocol conditions; ratio > 1 indicates VL dominance.

Athlete	Sport	*n* Cond.	VL/VM Ratio	Dominant Muscle
S1	Basketball	4	1.94 ± 0.78	VL
S2	Basketball	4	1.70 ± 0.51	VL
S3	Basketball	4	0.85 ± 0.07	VM
S4	Basketball	3	1.57 ± 0.34	VL
S5	Basketball	4	1.35 ± 0.50	VL
S6	Basketball	4	1.00 ± 0.56	Balanced
All	—	23	1.40 ± 0.61	VL (16/23)

## Data Availability

The data presented in this study are available from the corresponding author upon reasonable request.

## Supplementary Materials

The following supporting information can be downloaded at: https://www.mdpi.com/article/10.3390/jfmk11040395/s1, Table S1: Complete per-measurement sEMG fatigue-trend results (57 measurements; 8 athletes, 16 limbs).
