# Supplementary material for: Application of Artificial Intelligence and Surface Electromyography in the Assessment of Quadriceps Neuromuscular Fatigue and Functional Risk of Knee Injury in Professional Basketball Players: A Pilot Study
Source: J Funct Morphol Kinesiol. 2026 Oct 1;11(4):395. doi: 10.3390/jfmk11040395 (PMC13692500; doi:10.3390/jfmk11040395)
Supplement: Supplementary file 1 [file jfmk-11-00395-s001.zip › jfmk-4574202-supplementary.pdf]

Supplementary Materials

Artificial Intelligence and Surface Electromyography for Assessing Quadriceps Neuromuscular Fatigue and Knee Injury Risk in Professional Basketball Players: A Pilot Study

Đorđe Kosanić, Nemanja Marković, Radivoje Radaković, Nenad Filipović and Elvis Mahmutović

Table S1. Complete per-measurement sEMG fatigue-trend results (57 measurements; 8 athletes, 16 limbs). S1–S6 extended cohort, S7–S8 initial pilot. Protocol: Iso = isometric, Dyn = isotonic 30-rep. Muscle: VL/VM, or "–" for single-channel pilot recordings. Slopes per second; p from the trend test; Sig = fatigue signature (RMS↑ & MNF↓).

#	S	Protocol	Muscle	Leg	Dur (s)	RMS slope (μV·s⁻¹)	RMS p	MNF slope (Hz·s⁻¹)	MNF p	Sig
1	S1	Iso	VL	Right	46	+0.187	4e-05	+0.052	0.098	–
2	S1	Iso	VL	Left	46	+0.135	2e-08	-0.032	0.284	Yes
3	S1	Iso	VM	Right	46	-0.164	5e-10	-0.034	0.338	–
4	S1	Iso	VM	Left	53	+0.051	1e-03	-0.166	0.002	Yes
5	S1	Dyn	VL	Right	39	+0.022	0.816	-0.035	0.653	Yes
6	S1	Dyn	VL	Left	29	-0.060	0.752	-0.193	0.078	–
7	S1	Dyn	VM	Right	47	+0.275	2e-05	-0.043	0.414	Yes
8	S1	Dyn	VM	Left	35	+0.161	0.286	-0.015	0.888	Yes
9	S2	Iso	VL	Right	44	+0.004	0.912	-0.004	0.923	Yes
10	S2	Iso	VL	Left	44	+0.817	3e-15	-0.025	0.437	Yes
11	S2	Iso	VM	Right	46	+0.156	8e-06	-0.138	6e-05	Yes
12	S2	Iso	VM	Left	46	+0.133	8e-11	-0.106	2e-04	Yes
13	S2	Dyn	VL	Right	30	+0.081	0.549	-0.129	0.192	Yes
14	S2	Dyn	VL	Left	32	+0.668	4e-05	+0.092	0.587	–
15	S2	Dyn	VM	Right	40	+0.221	4e-04	-0.088	0.070	Yes
16	S2	Dyn	VM	Left	32	+0.153	0.018	-0.065	0.297	Yes
17	S3	Iso	VL	Right	44	+0.066	0.002	-0.099	0.003	Yes
18	S3	Iso	VL	Left	48	+0.170	7e-19	+0.019	0.575	–
19	S3	Iso	VM	Right	46	+0.112	5e-06	+0.008	0.778	–
20	S3	Iso	VM	Left	52	+0.228	9e-15	+0.102	0.013	–
21	S3	Dyn	VL	Right	32	+0.267	9e-07	-0.088	0.248	Yes
22	S3	Dyn	VL	Left	34	+0.292	3e-11	+0.309	3e-04	–
23	S3	Dyn	VM	Right	34	+0.407	3e-13	-0.053	0.353	Yes
24	S3	Dyn	VM	Left	28	+0.725	3e-16	-0.121	0.298	Yes
25	S4	Iso	VL	Left	45	-0.089	0.081	+0.099	0.002	–
26	S4	Iso	VM	Right	46	-0.020	0.609	+0.160	2e-05	–
27	S4	Iso	VM	Left	46	+0.006	0.745	+0.266	2e-08	–
28	S4	Dyn	VL	Right	30	+0.572	1e-04	+0.210	0.021	–
29	S4	Dyn	VL	Left	28	+0.386	0.104	-0.137	0.134	Yes
30	S4	Dyn	VM	Right	30	+0.378	8e-06	+0.123	0.139	–

31	S4	Dyn	VM	Left	31	+0.147	0.505	-0.054	0.524	Yes
32	S5	Iso	VL	Right	46	+0.036	0.439	-0.009	0.734	Yes
33	S5	Iso	VL	Left	45	+0.373	2e-10	-0.011	0.678	Yes
34	S5	Iso	VM	Right	45	+0.121	0.009	-0.145	0.003	Yes
35	S5	Iso	VM	Left	45	+0.003	0.941	+0.011	0.791	–
36	S5	Dyn	VL	Right	30	+0.343	0.008	-0.135	0.064	Yes
37	S5	Dyn	VL	Left	30	+0.910	3e-08	-0.095	0.233	Yes
38	S5	Dyn	VM	Right	30	+0.477	0.004	+0.020	0.847	–
39	S5	Dyn	VM	Left	30	-0.150	0.372	-0.295	5e-04	–
40	S6	Iso	VL	Right	45	+0.061	0.031	-0.010	0.750	Yes
41	S6	Iso	VL	Left	45	+0.217	2e-04	+0.031	0.496	–
42	S6	Iso	VM	Right	40	+0.144	0.156	+0.021	0.598	–
43	S6	Iso	VM	Left	44	+0.363	8e-19	+0.231	3e-07	–
44	S6	Dyn	VL	Right	30	+0.616	2e-05	+0.102	0.147	–
45	S6	Dyn	VL	Left	31	+0.409	8e-05	+0.135	0.018	–
46	S6	Dyn	VM	Right	30	-0.231	0.530	-0.071	0.213	–
47	S6	Dyn	VM	Left	30	+0.407	0.001	+0.092	0.247	–
48	S7	Iso	–	Bilat	66	+0.103	9e-05	-0.060	0.010	Yes
49	S7	Iso	–	Bilat	68	+0.291	2e-22	-0.105	7e-05	Yes
50	S7	Iso	–	Right	64	+0.177	7e-09	+0.005	0.850	–
51	S7	Iso	–	Right	61	+0.230	1e-08	-0.037	0.077	Yes
52	S7	Iso	–	Left	63	+0.132	0.003	-0.090	3e-04	Yes
53	S8	Iso	–	Right	61	-0.502	4e-13	-0.218	3e-22	–
54	S8	Iso	–	Left	80	+0.442	6e-15	-0.083	1e-05	Yes
55	S8	Dyn	–	Right	60	+0.690	2e-06	-0.303	2e-15	Yes
56	S8	Dyn	–	Left	64	+0.332	0.002	-0.212	1e-04	Yes
57	S8	Dyn	–	Left	58	+0.316	0.004	-0.162	1e-07	Yes

S = athlete identifier (S1–S6, extended cohort with separate VL and VM channels; S7–S8, initial pilot with a single composite quadriceps channel). Slopes are per second; p values are from the linear trend test. Sig = canonical fatigue signature (RMS increasing together with MNF decreasing). This table corresponds to Table 5 of the previous manuscript version and is referenced in Section 3.5 of the main text.
